# Supplementary material for: The clinical and molecular landscape of breast cancer in women of African and South Asian ancestry
Source: Nat Commun. 2025 May 20;16:4237. doi: 10.1038/s41467-025-59144-z (PMC12092682; doi:10.1038/s41467-025-59144-z)
Supplement: Supplementary file 3 — Description of Additional Supplementary Files [file 41467_2025_59144_MOESM3_ESM.pdf]

<u>Description of Additional Supplementary Files</u>

Supplementary Data 1. Statistics of clinical, tumour and molecular features in the Genomics England cohort: a. Descriptive statistics across the whole cohort (all p-values are two-sided, and corrected for multiple comparisons). **b.** Fits from the regression modelling comparing EUR/AFR and EUR/SAS cohorts within Genomics England (all p-values are two-sided from the relevant generalised linear model and not corrected for multiple comparisons) **c.** Fits from the regression modelling comparing EUR/AFR and EUR/SAS cohorts within Genomics England (split into under 50 and 50 and over cohorts) (all p-values are two-sided from the relevant generalised linear model and not corrected for multiple comparisons): **i.** Under 50; **ii.** 50 or over.

Supplementary Data 2. Statistics of clinical and tumour features of the TCGA cohort: a. Descriptive statistics across the whole cohort (all p-values are two-sided, and corrected for multiple comparisons). **b.** Fits from the regression modelling comparing EUR/AFR cohorts within TCGA (all p-values are two-sided and from the relevant generalised linear model and are not corrected for multiple comparisons).

Supplementary Data 3. Likelihood ratio tests of full model (gAncestry (EUR, AFR, SAS) and IMD) against basic model (gAncestry (EUR, AFR, SAS) alone) for demographic factors in Genomics England cohort: all p-values from the relevant generalised linear model are two-sided and uncorrected for multiple comparisons.

Supplementary Data 4. Descriptive statistics of clinical, tumour and molecular features across IMD quintiles for Genomics England EUR cohort: all p-values from the relevant test are two-sided and corrected for multiple comparisons.

Supplementary Data 5. Statistics of clinical and tumour features of the BCN Biobank-Barts cohort (excluding patients dually consented with Genomic England): a. Descriptive statistics across whole cohort (all p-values are two-sided, and corrected for multiple comparisons). **b.** Fits from regression

models comparing White/Black and White/Asian cohorts within the BCN Biobank (all p-values are two-sided, and not corrected for multiple comparisons).

Supplementary Data 6. Differentially mutated somatic genes in the Genomics England AFR and SAS cohorts. Differentially-mutated genes arise from the logistic model and the 2% threshold model (odds ratios and two-sided adjusted p-values only shown where significance was achieved in logistic model).

Supplementary Data 7. Differentially mutated somatic variants in the Genomics England AFR and SAS cohorts. Differentially-mutated variants arise from the logistic model and the 2% threshold model (odds ratios and two-sided adjusted p-values only shown where significance was achieved in logistic model).

Supplementary Data 8. Differentially mutated germline genes: a. Germline variants differentially mutated (two-sided logistic regression unadjusted p-value<0.05) in EUR v AFR in the Genomics England and TCGA cohorts. **b.** Germline variants differentially mutated (two-sided logistic regression unadjusted p-value<0.05) in EUR v SAS in the Genomics England and in the G&H case:control study.

Supplementary Data 9. Variant signatures in the Genomics England gAncestry cohorts: a. Single base substitution signatures (p-values are two-sided from Fisher exact test). Those in grey are (at most) only present in EUR cohort. **b.** Double base substitution signatures (p-values are two-sided from Fisher exact test). Those in grey are (at most) only present in EUR cohort. **c.** Rearrangement signatures (p-values are two-sided from Fisher exact test). Those in grey are (at most) only present in EUR cohort.

Supplementary Data 10. HRDetect and gAncestry in the Genomics England cohorts: a. Number of patients with HRDetect-defined HR deficiency in each cohort. **b.** HRDetect contribution scores (p-values by two-sided Wilcoxon rank-sum test).
