# Supplementary material for: The clinical and molecular landscape of breast cancer in women of African and South Asian ancestry
Source: Nat Commun. 2025 May 20;16:4237. doi: 10.1038/s41467-025-59144-z (PMC12092682; doi:10.1038/s41467-025-59144-z)
Supplement: Supplementary file 5 — Reporting Summary [file 41467_2025_59144_MOESM5_ESM.pdf]

nature portfolio

Corresponding author(s): Claude Chelala

Last updated by author(s): 4th April 2025

Reporting Summary

Nature Portfolio wishes to improve the reproducibility of the work that we publish. This form provides structure for consistency and transparency in reporting. For further information on Nature Portfolio policies, see our Editorial Policies and the Editorial Policy Checklist.

Statistics

For all statistical analyses, confirm that the following items are present in the figure legend, table legend, main text, or Methods section.

n/a	Confirmed	
☐	☒	The exact sample size (*n*) for each experimental group/condition, given as a discrete number and unit of measurement
☐	☒	A statement on whether measurements were taken from distinct samples or whether the same sample was measured repeatedly
☐	☒	The statistical test(s) used AND whether they are one- or two-sided<br>*Only common tests should be described solely by name; describe more complex techniques in the Methods section.*
☐	☒	A description of all covariates tested
☐	☒	A description of any assumptions or corrections, such as tests of normality and adjustment for multiple comparisons
☐	☒	A full description of the statistical parameters including central tendency (e.g. means) or other basic estimates (e.g. regression coefficient) AND variation (e.g. standard deviation) or associated estimates of uncertainty (e.g. confidence intervals)
☐	☒	For null hypothesis testing, the test statistic (e.g. *F*, *t*, *r*) with confidence intervals, effect sizes, degrees of freedom and *P* value noted<br>*Give P values as exact values whenever suitable.*
☒	☐	For Bayesian analysis, information on the choice of priors and Markov chain Monte Carlo settings
☒	☐	For hierarchical and complex designs, identification of the appropriate level for tests and full reporting of outcomes
☒	☐	Estimates of effect sizes (e.g. Cohen's *d*, Pearson's *r*), indicating how they were calculated

Our web collection on statistics for biologists contains articles on many of the points above.

Software and code

Policy information about availability of computer code

Data collection	R scripting language (base) with library Rlabkey (within Genomics England TRE)
Data analysis	R scripting language (with libraries: HRDetect, signature.tools.lib, Rlabkey, lmtest, arsenal, tidyverse; ggplot2 and forestplot for visualisation)

For manuscripts utilizing custom algorithms or software that are central to the research but not yet described in published literature, software must be made available to editors and reviewers. We strongly encourage code deposition in a community repository (e.g. GitHub). See the Nature Portfolio guidelines for submitting code & software for further information.

Data

Policy information about availability of data

All manuscripts must include a data availability statement. This statement should provide the following information, where applicable:

- Accession codes, unique identifiers, or web links for publicly available datasets
- A description of any restrictions on data availability
- For clinical datasets or third party data, please ensure that the statement adheres to our policy

Genomic and phenotypic data for the 100KGP study participants are available through the Genomics England Research Environment via application at https://www.genomicsengland.co.uk/join-us: approval to access the anonymised data through the Genomics England Trusted Research Environment requires a research project proposal, and mandatory training on information governance.
The clinical data and donor genetic ancestry calls for the TCGA cohort used in this study are available from the supplemental information (Table S1) of Carrot-Zhang

et al. (https://doi.org/10.1016/j.ccell.2020.04.012; Table S1 at https://ars.els-cdn.com/content/image/1-s2.0-S1535610820302117-mmc2.xlsx) Additional clinical and genomic data for the TCGA BRCA cohort are accessible from the Genomics Data Commons Data Portal (https://portal.gdc.cancer.gov/), with the clinical data openly downloadable; somatic data are downloadable following application to dbGaP (https://gdc.cancer.gov/access-data/obtaining-access-controlled-data, accession ID phs00178, https://www.ncbi.nlm.nih.gov/projects/gap/cgi-bin/study.cgi?study_id=phs000178.v11.p8) for the controlled somatic variation data.
Germline TCGA-BRCA data is available from the PanCanAtlas, again through applying through dbGaP for the controlled data in the GDC portal under the same accession ID (phs00178).
Clinical data for BCN Biobank participants is available (anonymised) on application to the Biobank (https://breastcancernow.org/our-research/information-for-researchers/apply-to-our-biobank/how-to-apply-to-the-biobank, https://biobank.bcc.qmul.ac.uk/surveys/?s=KEAEJ4RFRH)
Clinical and molecular data from G&H is available through the Genes and Health Research Environment via application at https://www.genesandhealth.org/researchers/apply-for-access/: applicants are required to fill in a Data Access Agreement, complete mandatory information governance training and pay a small fee for access to the trusted research environment to examine individual-level data.
For the restricted data classes, summarised data can be exported from the trusted research environments for research purposes with restrictions to prevent the identification of individual participants through data linkage.

Research involving human participants, their data, or biological material

Policy information about studies with human participants or human data. See also policy information about sex, gender (identity/presentation), and sexual orientation and race, ethnicity and racism.

Reporting on sex and gender	All cohorts used in this paper were female. For Genomics England, sex was assigned by the karyotype of the respective germline sample. For TCGA, BCN Biobank and Genes and Health, gender was self-reported as no karyotyping information was available.
Reporting on race, ethnicity, or other socially relevant groupings	TCGA data used 'self-reported ethnicity' split into the main groups White, Black or African American, Asian or other, and '[self-reported] race' split into Hispanic and non-Hispanic.<br>Other data sets used self-reported ethnicity and ethnic subgroups as classified under the England and Wales Census 2021.
Population characteristics	All participants in this study had a primary disgnosis of breast cancer, aside from the sex and ancestry-matched control cohort taken from Genes and Health
Recruitment	We performed no recruitment ourselves; all recruitment was performed by the relevant study (TCGA, Breast Cancer Now Biobank, Genes and Health and Genomics England)
Ethics oversight	East of England - Cambridge Central Research Ethics Committee gave ethical approval for the Breast Cancer Now Biobank (REC reference: 23-EE-0229). Genomics England Clinical Interpretation Partnership (GECIP) gave approval for this work reference 643) to use Genomics England data. Genes & Health gave approval to this work reference S00087 to use the Genes & Health data. The Data Access Committee for The Cancer Genome Atlas (TCGA) gave approval for this work reference dbGaP project #15970 to use TCGA data.

Note that full information on the approval of the study protocol must also be provided in the manuscript.

Field-specific reporting

Please select the one below that is the best fit for your research. If you are not sure, read the appropriate sections before making your selection.

☒ Life sciences ☐ Behavioural & social sciences ☐ Ecological, evolutionary & environmental sciences

For a reference copy of the document with all sections, see nature.com/documents/nr-reporting-summary-flat.pdf

Life sciences study design

All studies must disclose on these points even when the disclosure is negative.

Sample size	The study dataset comprised 7.253 breast cancer patients from four cohorts - 3,334 from Genomics England, 2,479 from Barts Health NHS Trust patients within the BCN Biobank (BCN Biobank-Barts cohort), 1,076 from TCGA and 364 from Genes and Health. A control cohort of 17,626 ancestry and sex-matched non-breast cancer participants were also used from Genes and Health. Considering the exclusions (below) these were the largest available from the data sources
Data exclusions	In the cohorts, participants were excluded if they were not female (sex) or did not have primary breast cancer (for the breast cancer cohorts)
Replication	Results were replicated across the four cohorts of breast cancer as much as possible where similar attributes were available
Randomization	There was no randomization
Blinding	There was no randomization

Reporting for specific materials, systems and methods

We require information from authors about some types of materials, experimental systems and methods used in many studies. Here, indicate whether each material, system or method listed is relevant to your study. If you are not sure if a list item applies to your research, read the appropriate section before selecting a response.

Materials & experimental systems

n/a	Involved in the study
☒	☐ Antibodies
☒	☐ Eukaryotic cell lines
☒	☐ Palaeontology and archaeology
☒	☐ Animals and other organisms
☐	☒ Clinical data
☒	☐ Dual use research of concern
☒	☐ Plants

Methods

n/a	Involved in the study
☒	☐ ChIP-seq
☒	☐ Flow cytometry
☒	☐ MRI-based neuroimaging

Clinical data

Policy information about clinical studies

All manuscripts should comply with the ICMJE guidelines for publication of clinical research and a completed CONSORT checklist must be included with all submissions.

Clinical trial registration	This is not a clinical trial
Study protocol	The protocol for analysis is available in the Supplementary Methods
Data collection	Genomics England data release v17 (30th March 2023); BCN Biobank data extract of 27th April 2023; TCGA last accessed 24th July 2024; Genes and Health extraction date 23rd February 2024.
Outcomes	Survival data in v17 of Genomics England data release

Plants

Seed stocks	No plants used
Novel plant genotypes	No plants used
Authentication	No plants used
